# Wastewater Metagenomic Surveillance Reveals Socioeconomic Patterns of Pathogen Diversity and Antimicrobial Resistance in Nairobi, Kenya

**DOI:** 10.3390/antibiotics15090845

**Published:** 2026-08-31

**Authors:** Benard Mware, Gilbert Kibet-Rono, Kennedy Mwangi, Doreen Lugano, Shebbar Osiany, Edward Kiritu, Paul O. Dobi, Collins Muli, John Juma, Regina Njeru, Eunice M. Machuka, Bernard Bett, Ahmed Ogwell, Edward O. Abworo, Tulio de Oliveira, Dishon M. Muloi, Sofonías K. Tessema, Samuel O. Oyola

**Affiliations:** 1 International Livestock Research Institute, Uthiru, Naivasha Road, P.O. Box 30709, Nairobi 00100, Kenya; b.mware@cgiar.org (B.M.); g.kibet@cgiar.org (G.K.-R.); k.mwangi@cgiar.org (K.M.); dlugano@kemri-wellcome.org (D.L.); s.osiany@cgiar.org (S.O.); e.kiritu@cgiar.org (E.K.); p.dobi@cgiar.org (P.O.D.); c.muli@cgiar.org (C.M.); j.juma@cgiar.org (J.J.); r.njeru@cgiar.org (R.N.); e.machuka@cgiar.org (E.M.M.); b.bett@cgiar.org (B.B.); e.okoth@cgiar.org (E.O.A.); d.muloi@cgiar.org (D.M.M.); 2 Africa Centres for Disease Control and Prevention, African Union Headquarters, Addis Ababa P.O. Box 200050, Ethiopia; ogwellouma@gmail.com (A.O.); sofonias.tessema@gatesfoundation.org (S.K.T.); 3 Centre for Epidemic Response and Innovation (CERI), School of Data Science and Computational Thinking, Stellenbosch University, Francie van Zijl Drive, Tygerberg, Stellenbosch, Cape Town 7505, South Africa; tulio@sun.ac.za

**Keywords:** genomic epidemiology, metagenomics, wastewater-based environmental surveillance (WES), antimicrobial resistance genes (ARGs), antimicrobial resistance (AMR), genomic abundance, resistome, ESKAPE pathogens

## Abstract

**Background:** Wastewater and Environmental Surveillance (WES) has become a useful public health tool as an early-warning system revealing emergence/re-emergence of pathogenic diseases and spread of antimicrobial resistance (AMR). We characterized the taxonomic composition, relative abundance, and antibiotic resistance genes (ARGs) of wastewater-identified bacterial pathogens across socioeconomically and epidemiologically diverse sewerage catchments in Nairobi, Kenya—a key East African urban city representing a low- and middle-income country (LMIC). **Results:** Metagenomic analysis of Nairobi’s wastewater revealed distinct bacterial and antimicrobial resistance (AMR) profiles. *Campylobacteraceae* (52.5% ± 17.9%) and *Bacteroidaceae* (19.8% ± 9.9%) dominated the communities. While *Arcobacter cryaerophilus* was ubiquitous, *Bacteroides fragilis* and *A. suis* abundances varied by neighborhood socioeconomic status. We detected critical clinical pathogens—including *Escherichia coli*, *Vibrio cholerae*, *Mycobacterium tuberculosis*, and the ESKAPE species—alongside 207 distinct ARGs conferring resistance to 11 antibiotic classes. Both taxonomic and ARG compositions showed high spatial heterogeneity, with maximum variation in low-income areas. Temporal analysis captured shifting pathogen dynamics, and metagenomic abundances for *Vibrio cholerae* and *Klebsiella pneumoniae* were validated via qPCR. **Conclusions:** Our findings underscore the utility of WES as a scalable, non-invasive public health tool. By capturing community-level pathogen composition and AMR dynamics, WES bypasses the limitations of clinical diagnostic access, providing a vital early-warning system for underserved urban populations. To maximize its public health utility, environmental genomic signals must serve as actionable triggers for coordinated One Health responses, including targeted clinical diagnostics, localized antimicrobial stewardship reviews, proactive risk communication, and prioritized sanitation infrastructure upgrades.

## 1. Introduction

Wastewater-based epidemiology (WBE) has emerged as a powerful approach for monitoring infectious disease transmission and antimicrobial resistance (AMR) at the community level. By surveilling pathogens, resistance genes, and antimicrobial residues in sewage, WBE can generate near-real-time, population-level data that complement and, in some cases, overcome limitations of traditional clinical surveillance, such as under-reporting, delayed case detection, and inequities in healthcare access [1]. Recent advances in sampling strategies, concentration methods, and molecular techniques, including qPCR and metagenomics, have expanded the scope and sensitivity of WBE while also highlighting key challenges around data normalization, interpretation, and ethical use of population-scale information. As emphasized by Sims and Kasprzyk-Hordern [1], integrating WBE with clinical and environmental datasets offers substantial potential for earlier outbreak detection, tracking the spread of emerging pathogens and resistance determinants, and guiding targeted public health interventions, positioning WBE as a critical component of modern infectious disease and AMR surveillance frameworks. Timely and effective disease surveillance is critical for informing public health interventions and preventing widespread outbreaks. WES has emerged as a valuable complement to conventional surveillance systems, offering a cost-effective and non-invasive means to monitor population health and serve as an early-warning system for disease emergence [2,3].

By analyzing wastewater from sewer catchments, WES enables detection of pathogens and ARGs at the community level, providing insights into both pathogen circulation and AMR dynamics. The convergence of diverse microbial communities in wastewater, combined with selective pressures from antibiotics and disinfectants, may also promote the emergence and horizontal transfer of resistance genes [4,5,6].

While wastewater-based genomic surveillance is increasingly recognized as a scalable method for pathogen and AMR detection, limited data exist on its application in urban African settings or its integration with socio-economic determinants. This study addresses this gap by applying high-throughput metagenomic sequencing to characterize bacterial community composition, diversity, abundance, and associated ARGs across Nairobi’s sewerage network. Populations were stratified into low-, middle-, and high-income groups to evaluate how socio-economic context influences the distribution of pathogenic and antimicrobial-resistant bacteria in an urban environment.

Our study provides a comprehensive application of metagenomic surveillance in a rapidly urbanizing African city. We confirmed the presence of multidrug-resistant ESKAPE pathogens and other high-risk bacteria in Nairobi’s wastewater and demonstrated the reliability of integrating metagenomics with quantitative PCR for pathogen validation. The findings highlight how socio-economic disparities shape microbial diversity and AMR gene distribution. More broadly, these findings establish WES as a viable and equity-sensitive public health tool that can be harnessed as an early-warning system, particularly in low-resource settings. Given that Nairobi exemplifies many rapidly urbanizing environments in low- and middle-income countries—characterized by dense populations; inadequate water, sanitation, and hygiene infrastructure; and limited surveillance capacity—our approach and findings are both relevant and generalizable to similar global contexts.

## 2. Results

**Figure 1:**
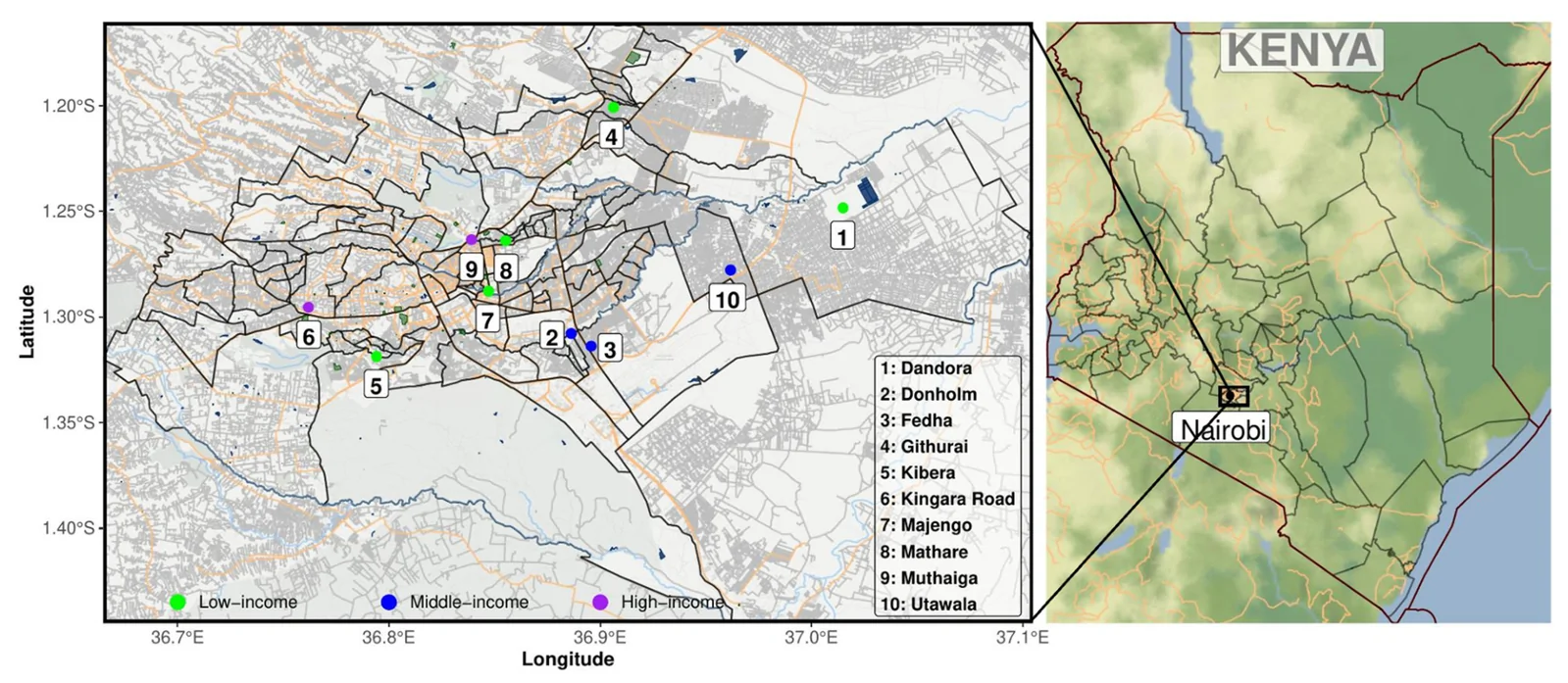
Map of Nairobi, Kenya, showing the closed sewerage network and the ten sampling points.

**Figure 2:**
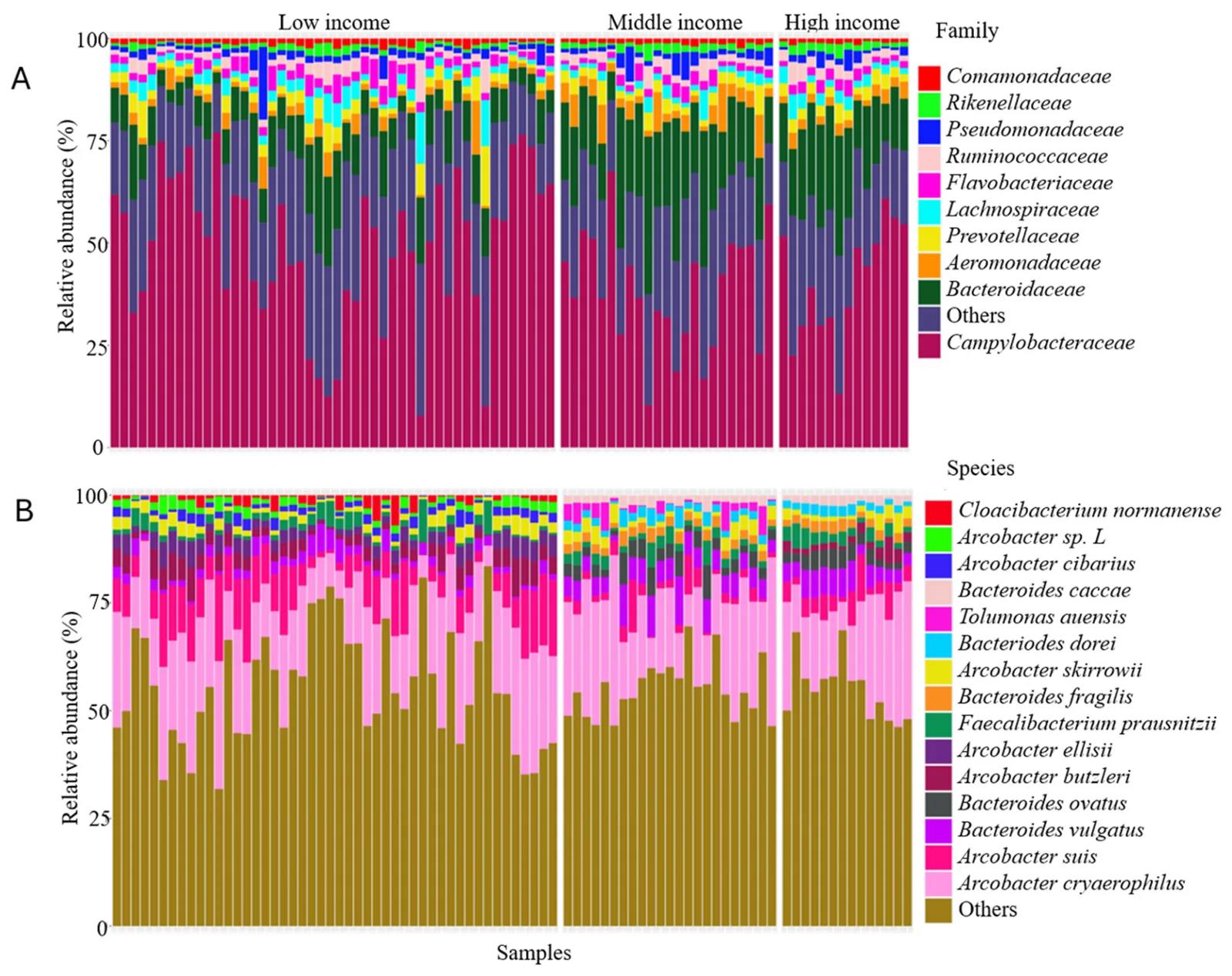
Relative abundance of bacterial pathogens in wastewater samples. (**A**) Stacked bar plot showing the top 10 bacterial families identified in wastewater samples collected in Nairobi. (**B**) Compositional relative abundance of bacterial species taxonomically identified in wastewater samples collected in Nairobi.

This study characterized the bacterial profiles across Nairobi’s sewerage network (Figure 1), stratified by income levels (Table 1), to elucidate patterns of pathogen distribution and their potential public health implications. Analysis revealed Campylobacteraceae as the dominant bacterial family (52.52% ± 17.92%) (Figure 2A), followed by Bacteroidaceae (19.78% ± 9.96%), Pseudomonadaceae (5.01% ± 4.35%), Prevotellaceae (4.40% ± 2.78%), Aeromonadaceae (4.10% ± 3.11%), Lachnospiraceae (3.95% ± 2.58%), Ruminococcaceae (3.38% ± 2.08%), and Flavobacteriaceae (3.05% ± 1.57%). Within the Campylobacteraceae, seven *Arcobacter* species were identified, with *A. cryaerophilus* (21.01% ± 7.39%) being the most prevalent, followed by *A. suis* (4.06% ± 2.17%), *A. butzleri* (2.43% ± 1.60%), and *A. skirrowii* (2.34% ± 1.37%).

Across all income levels (socio-economic zones), *A. cryaerophilus* remained the predominant species (22.30% ± 9.48%), accompanied by *Bacteroides vulgatus* (5.26% ± 2.54%), *B. ovatus* (3.60% ± 1.86%), *A. suis* (2.80% ± 3.33%), *A. skirrowii* (2.44% ± 1.47%), *B. fragilis* (2.17% ± 0.42%), *Tolumonas auensis* (2.13% ± 2.02%), *B. caccae* (2.07% ± 0.82%), and *Faecalibacterium prausnitzii* (2.06% ± 1.41%) (Figure 2B). Significant differences in microbial composition were observed across the different income-level strata. While *A. cryaerophilus* exhibited relatively uniform distribution across all zones, *A. suis* was markedly more abundant in low-income areas compared to middle- and high-income zones (Figure 2B). Conversely, most *Bacteroides* species, including *B. fragilis* and *B. ovatus*, were enriched in high- and middle-income zones but comparatively scarce in low-income areas.

**Figure 3:**
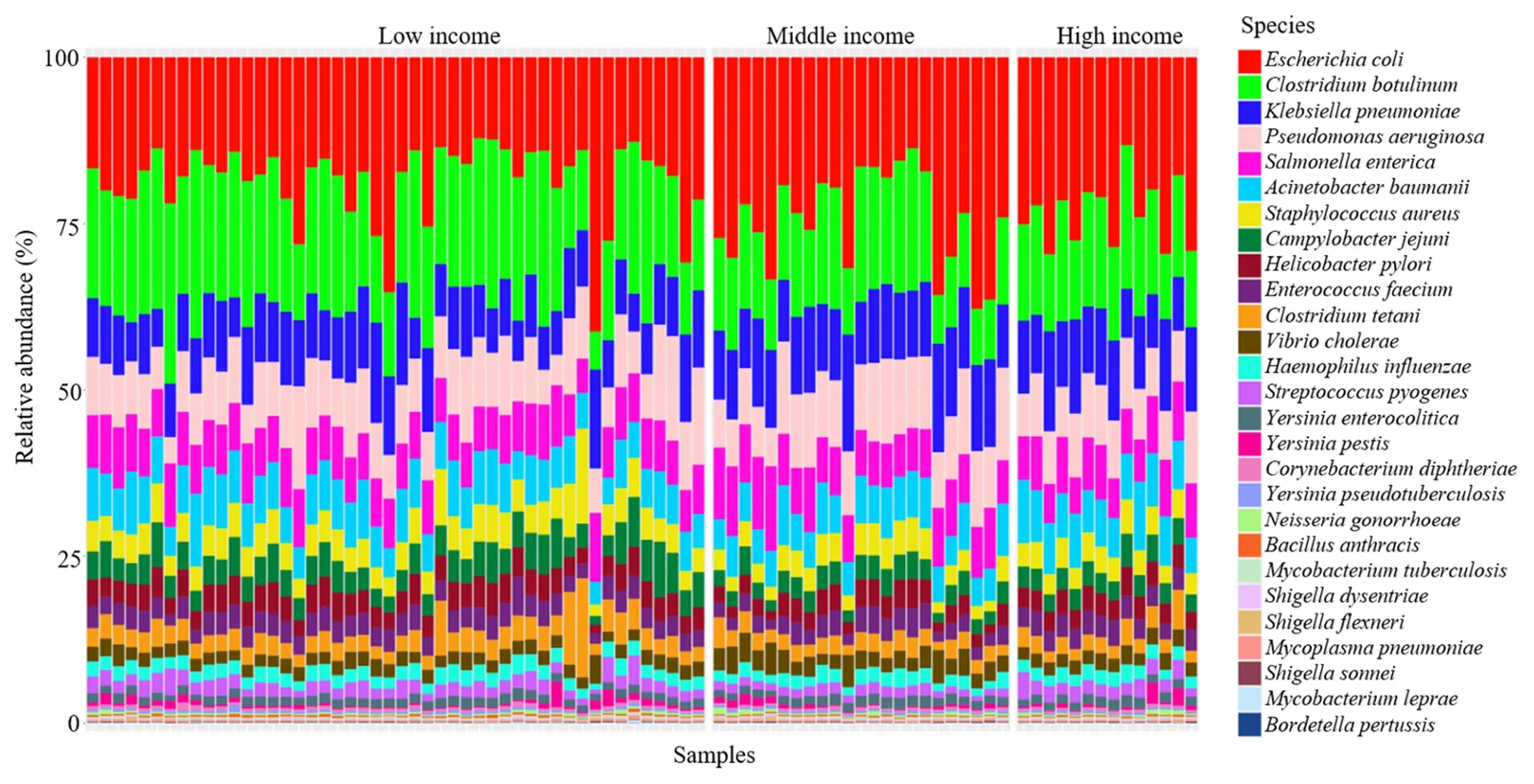
Relative abundance of bacterial pathogens. The stacked bar graph shows the relative abundance of bacterial species of public health interest classified taxonomically across wastewater sampling sites, representing populations among socio-economic settings (low-, middle-, and high-income) in Nairobi.

The metagenomic analysis revealed the presence of multiple bacterial pathogens of significant public health concern (Figure 3), including *Escherichia coli*, *Staphylococcus* spp., *Streptococcus* spp., *Mycobacterium* spp., *Clostridium botulinum*, and *Vibrio cholerae*. Importantly, several zoonotic pathogens were identified, such as *Bacillus anthracis*, *Mycobacterium avium*, *Mycobacterium tuberculosis*, and *Campylobacter* spp., highlighting the interconnectedness of human and animal health within urban environments.

Of particular interest was the detection of the ESKAPE group of pathogens—*Enterococcus faecium*, *Staphylococcus aureus*, *Klebsiella pneumoniae*, *Acinetobacter baumannii*, *Pseudomonas aeruginosa*, and *Enterobacter* spp.—which are globally recognized for their role in antimicrobial resistance (AMR) and nosocomial infections [7].

**Figure 4:**
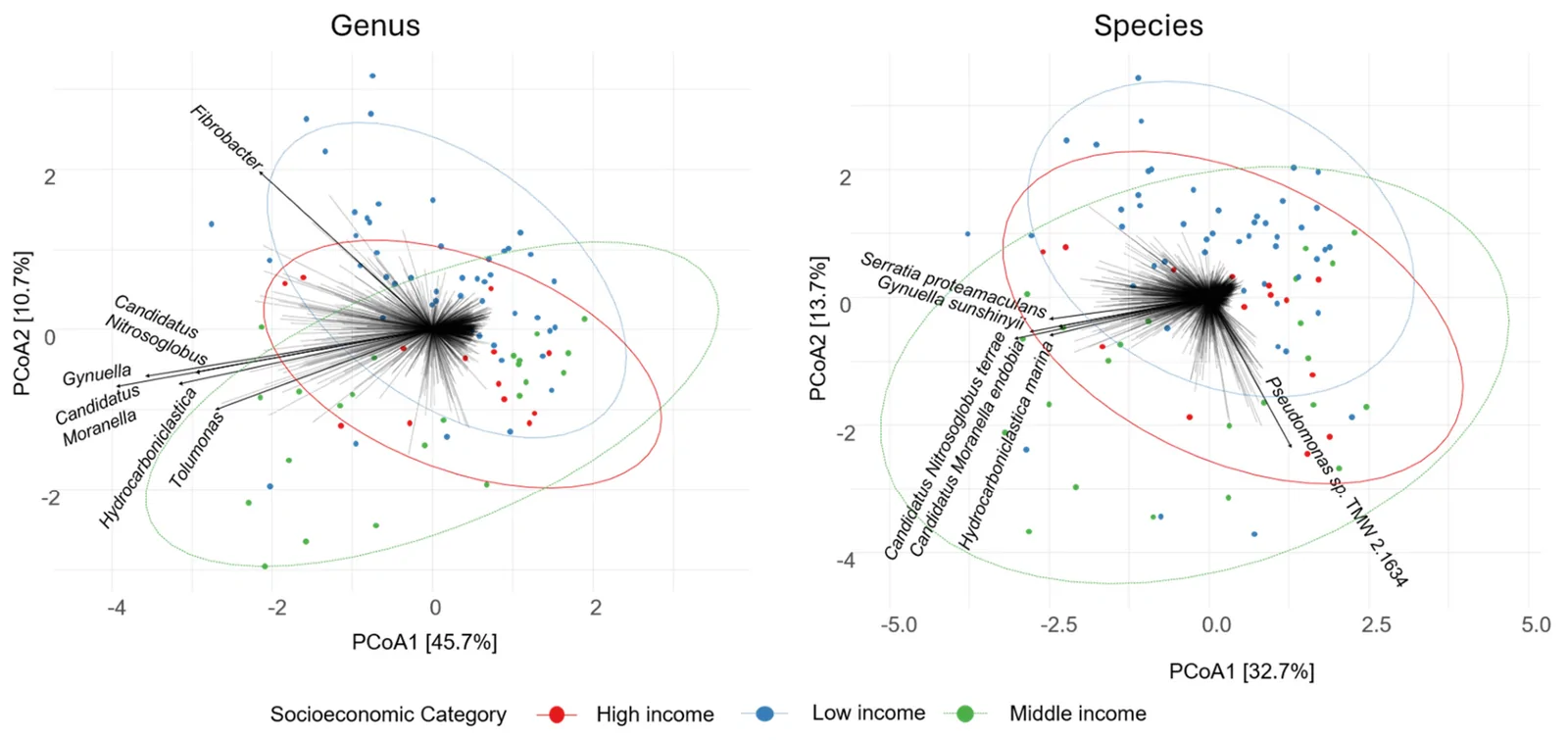
Principal Coordinate Analysis (PCoA) using Aitchison distance at the genus (**left**) and species (**right**) levels across different sampling time points in various socio-economic populations. The top six genera (**left**) and species (**right**) contributing to the differences between socio-economic groups are highlighted. Prior to calculation of the Aitchison distance, we applied centred log-ratio (CLR) transformation. Statistically significant differences were observed between low income versus middle income at both genus (*p* = 0.006) and species levels (*p* = 0.003), and between low income and high income also at genus (*p* = 0.027) and species (*p* = 0.015) taxa levels.

Using principal coordinate analysis (PCoA) based on Aitchison distance, we observed significant dissimilarities in bacterial community structure. Variability in diversity and relative abundance ranged from 10.7% to 45.7% at the genus level (n = 1126) and from 13.7% to 32.7% at the species level (n = 3867) across the sampling sites representing low-, middle-, and high-income socioeconomic strata (Figure 4).

Notably, *Fibrobacter* spp. was most abundant in low-income areas, suggesting a microbial signature linked to environmental or lifestyle factors unique to these communities. In middle-income areas, genera such as *Candidafus*, *Tolumonas*, *Gynuella*, and *Hydrocarboniclastica* were most prevalent. In contrast, *Pseudomonas* spp. dominated the bacterial composition of high-income areas (Figure 4).

**Figure 5:**
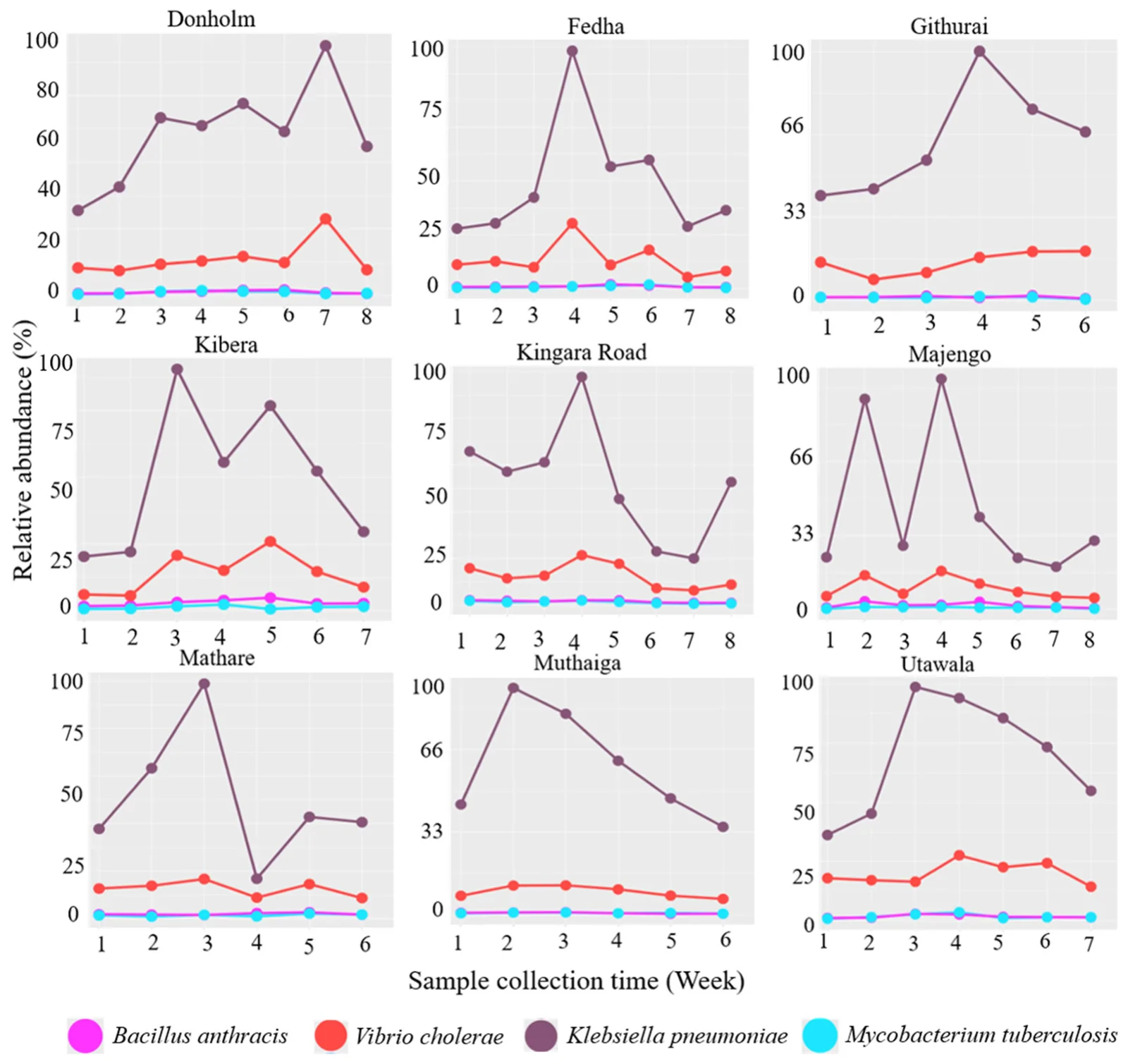
Spatiotemporal relative abundance of selected pathogens. Abundance and temporal fluctuations of pathogenic bacterial species across different sampling time points and sites in Nairobi City. A noticeable gradual fluctuation in abundance, peaking for *Klebsiella pneumoniae*, is observed for various pathogens at different sampling sites, reflecting distinct epidemiological population zones.

We analyzed temporal changes in the bacterial pathogen profiles (Figure 5) and observed dynamic fluctuations in the relative abundance of several pathogenic species over the study period. The results reveal marked temporal variation, characterized by distinct peaks and troughs in pathogen abundance across populations representing different socioeconomic (income) groups. The Dandora sampling site, located at the wastewater treatment plant (WWTP) and representing a convergence point for multiple sewer lines, exhibited a composite pathogen profile reflecting the cumulative microbial signatures from across the catchment area (Figure S3).

**Figure 6:**
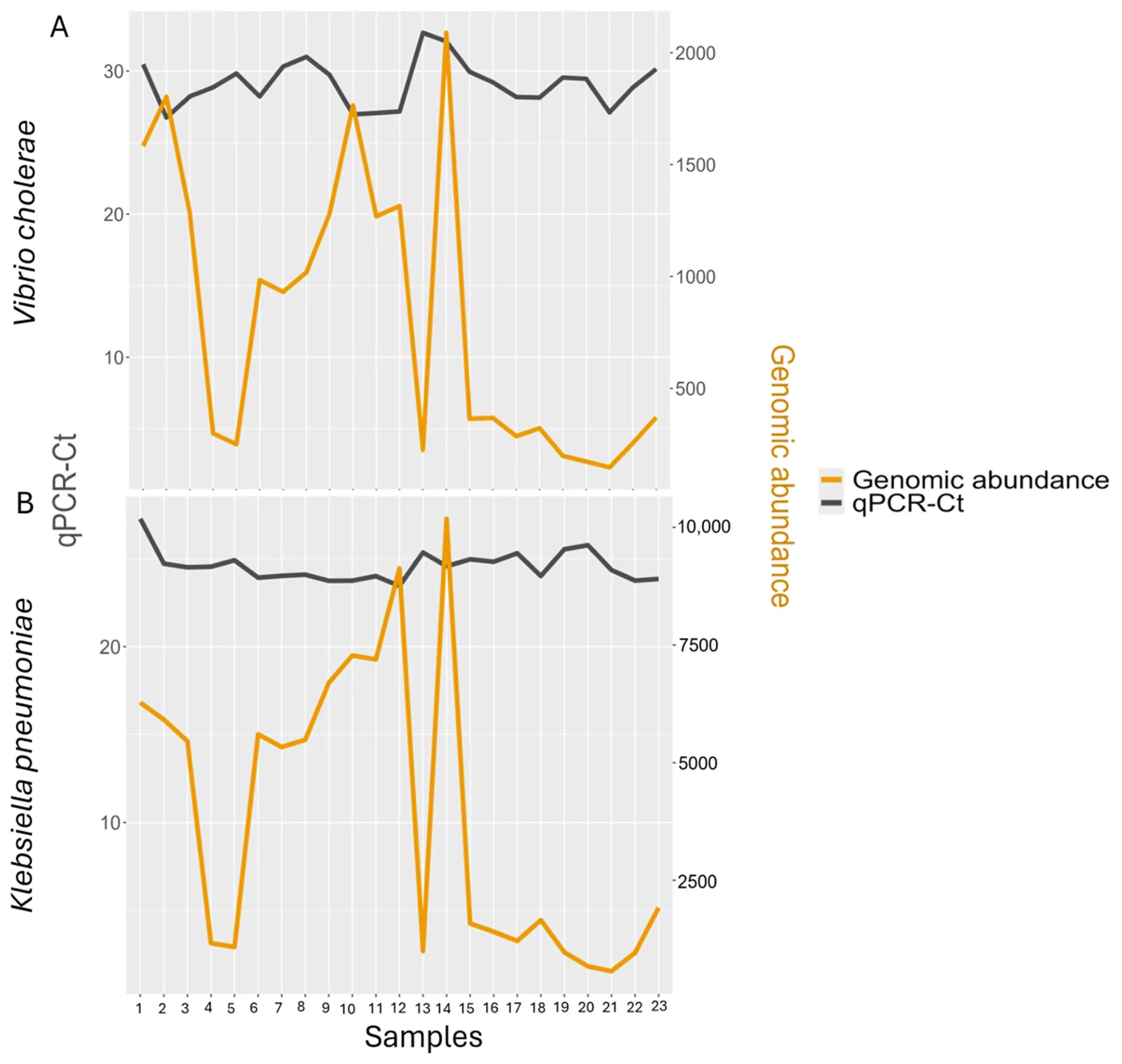
Correlation of genomic abundance and qPCR cycle threshold (Ct) values tested for *Vibrio cholerae* and *Klebsiella pneumoniae* from samples collected from the wastewater treatment site (Dandora) across different time points.

To validate the taxonomic classification and abundance of pathogens, molecular analysis was conducted using a published set of quantitative polymerase chain reaction (qPCR) primers (Table S1) targeting *Vibrio cholerae* and *Klebsiella pneumoniae* as pathogens of public health concern. This allowed for the detection and quantification of the pathogen in the various samples, and the results were compared to genomic abundance (Figure 6). The validation process confirmed metagenomic pathogen detection, showing a correlation between qPCR data and genomic abundance (Figure 6 and Figure S1). *V. cholerae* and *K. pneumoniae* qPCR data (Ct values) demonstrated the expected inverse relationship, where lower Ct values corresponded to higher genomic abundance.

**Figure 7:**
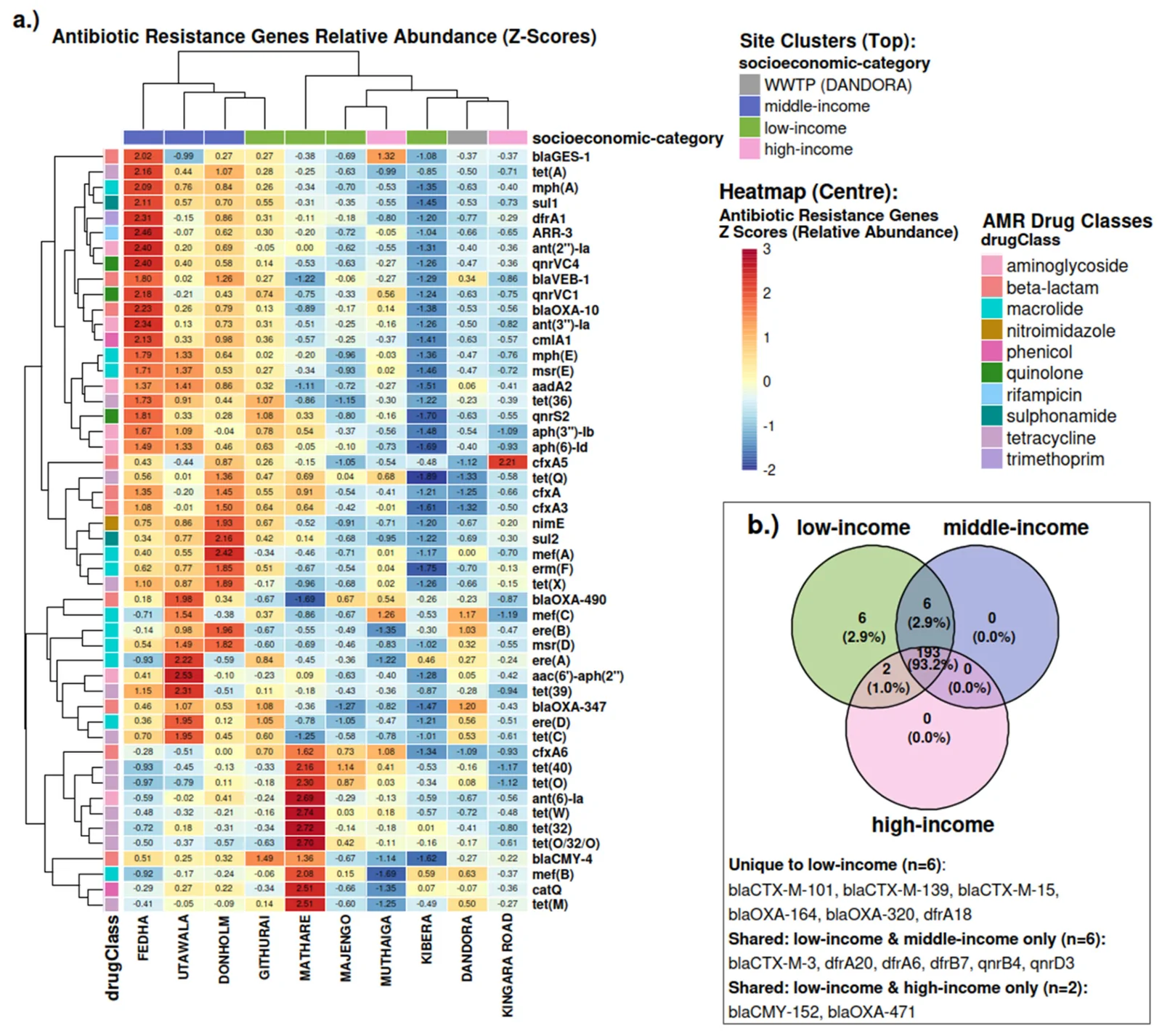
(**a**) Heatmap of 50 most abundant Antibiotic Resistance Genes (ARGs) illustrating the relative abundance of antibiotic resistance across sites. The numbers in the heatmap represent Z-scores calculated from the relative abundance (RA) data, indicating the number of standard deviations (+ or −) above or below the mean RA for each AMR gene across sites. The Z-score is calculated as: z = (x − μ)/σ, where *x* is the relative abundance, *μ* is the mean, and *σ* is the standard deviation of that specific ARG across sites. The dendrograms at the top and left represent hierarchical clustering of ARGs and sampling sites based on similarities in ARG occurrence patterns. Sites are categorized according to socio-economic status, with high-income sites shown in pink, low-income sites in green, and middle-income sites in blue. (**b**) Venn diagram illustrating the overlapping occurrence patterns of 207 predicted ARGs across the socio-economic categories; 6 (2.9%) ARGs were unique to low-income sites, and 6 (2.9%) and 2 (1.0%) were shared between low-income and middle-income sites and low-income and high-income sites, respectively. The rest of the genes were shared across the socioeconomic categories.

Antimicrobial resistance represents a major global health threat, being responsible for 1.27 million global deaths and contributing to 4.95 million deaths in 2019 [8]. In an attempt to investigate the relationship between socioeconomic status and the prevalence of priority bacterial pathogens, along with associated antibiotic resistance genes, we identified 207 resistome distinct ARGs associated with 11 antibiotic drug classes (Figure 7). These comprised beta-lactam (n = 66), aminoglycoside (n = 30), trimethoprim (n = 26), tetracycline (n = 24), macrolide (n = 24), quinolone (n = 12), phenicol (n = 9), nitroimidazole (n = 6), sulphonamide (n = 4), rifampicin (n = 4) and colistin (n = 2).

**Figure 8:**
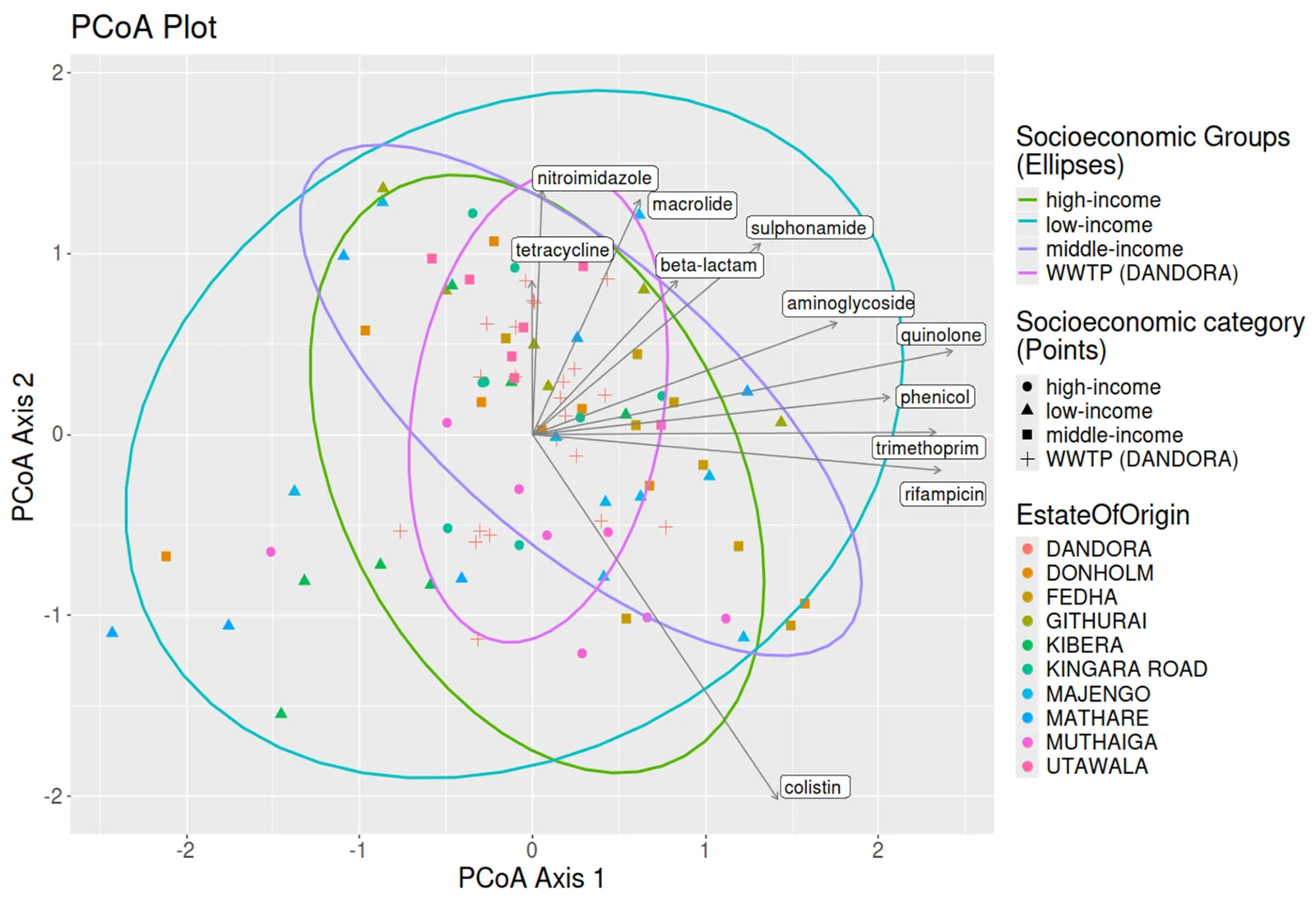
Principal Coordinate Analysis (PCoA) of antibiotic resistance profiles based on Aitchison distance, illustrating the clustering of resistance classes across sampling sites and socio-economic populations in Nairobi City. Points represent individual samples, coloured by estate of origin and shaped by socio-economic category (high-income, low-income, middle-income, and Central-waste water treatment plant (WWTP-DANDORA)). Ellipses denote 95% confidence intervals for each socio-economic group. Vector arrows indicate the direction and relative contribution of individual antibiotic classes (e.g., beta-lactam, sulphonamide, aminoglycoside, quinolone, colistin) to sample ordination along PCoA Axes 1 and 2.

PCoA (Figure 8) reveals the dissimilarity between samples based on the relative abundance composition of drug-class resistance. At a 95% confidence interval, ellipses group the samples into clusters according to their socio-economic classification. The analysis indicates greater variation in samples from low-income sites. However, there is noticeable overlap in the ARG composition across all socio-economic categories, with only marginal variation observed.

Analysis of antimicrobial resistance (AMR) gene class and drug class composition across wastewater catchments serving low-, middle-, and high-income areas revealed a statistically significant association between socioeconomic category and resistome composition at the AMR gene class level. The relative abundances of the 50 most abundant ARGs across sampling sites are provided in Table S2. This effect was robust across two distance measures with different underlying assumptions—Bray–Curtis dissimilarity and Robust Aitchison distance, with the latter explicitly accounting for the compositional nature of relative abundance data [9]. Socioeconomic category explained approximately 10–14% of the variance in community composition (PERMANOVA, *p* = 0.001 for both combinations tested), after controlling for sampling week and sequencing batch. At the broader drug class level, the effect was less consistent: it was supported by Bray–Curtis dissimilarity (R^2^ ≈ 0.07–0.14, *p* ≤ 0.001) but not by Robust Aitchison distance (R^2^ ≈ 0.03–0.04, *p* = 0.32–0.37; Figure S2), possibly reflecting a disproportionate influence of the most abundant drug classes.

Post hoc pairwise comparisons at the AMR gene class level indicated that this overall association was driven primarily by the middle-income category, which differed significantly from each of the other categories (pairwise PERMANOVA, *p*.adj ≤ 0.02). High-income, low-income, and Dandora mixed/composite (Central-WWTP) catchment sites were not significantly distinguishable from one another. This pattern was consistent across both distance measures.

Notably, colistin resistance showed a distinct ordination pattern, with its vector oriented almost orthogonally to the other antibiotic classes along PCoA Axis 2 and aligning with samples from high-income catchments. This separation suggests that colistin resistance was distributed differently from the majority of resistance classes and was more characteristic of the high-income category, in contrast to the co-varying cluster of aminoglycoside, quinolone, phenicol, and sulphonamide resistance.

## 3. Discussion

WES provides a cost-effective strategy for real-time detection and profiling of biomarkers, offering insights into public health risks related to emerging infectious diseases, AMR, and exposure to pollutants [10,11]. This study aimed to generate data that supports the development of an effective wastewater surveillance system for health intelligence signals, including early-warning systems, to guide public health interventions. The increasing emergence of antibiotic resistance genes has significantly heightened microbial risks and disease burden globally [12].

In this study, we detected several bacterial pathogens of public health importance, particularly those in the ESKAPE group, known for their ability to develop multidrug resistance, posing substantial clinical and economic challenges [13]. The potential for these pathogens to evade treatment and enter downstream aquatic environments and sludge presents serious public health risks [14,15]. The detection of multi-drug resistant organisms further positions this dataset as a critical resource for downstream AMR analysis and risk assessment. These findings reinforce the value of metagenomic surveillance in urban wastewater systems for timely detection of emerging infectious threats, supporting public health preparedness through a One Health approach. The misuse of antibiotics, compounded by inadequate access to clean water, sanitation, hygiene, and healthcare infrastructure, exacerbates the spread of resistant pathogens. Socioeconomic factors, including living conditions and education, further increase the risk of acquiring antibiotic-resistant bacteria [16].

Other notable pathogens, including *Bacillus anthracis*, *Mycobacterium tuberculosis*, *Clostridium botulinum*, and *Vibrio cholerae*, exhibited varying abundance over time (Figure 5) and across different socio-economic groups. Further investigation is needed to explore whether these changes correlate with pre-outbreak infection thresholds within affected populations, potentially informing public health action. Temporal data on pathogen abundance, combined with socio-economic indicators and clinical infection data, could aid in establishing disease outbreak models and provide critical early-warning signals for timely public health responses. Previous research has established a correlation between low socioeconomic status and increased susceptibility to colonization or infection by antibiotic-resistant pathogens. Contributing factors include limited access to healthcare, lower educational attainment, reduced income, and higher levels of residential crowding—conditions that collectively elevate public health risks [16]. These findings suggest that socioeconomic status not only shapes pathogen diversity but may also influence exposure to antibiotic-resistant organisms, reinforcing the need for equitable public health strategies that integrate environmental surveillance data. These temporal dynamics can be modeled to monitor circulating pathogens within the population and serve as an early-warning system for timely public health responses.

A promising approach for bacterial detection involves targeting highly transcribed genes as signature sequences, detecting both DNA and RNA transcripts [17]. Real-time PCR, which amplifies both DNA and RNA with high sensitivity, can allow detection of low quantities of pathogens, making it an effective tool for ultrasensitive bacterial detection. In this study, real-time PCR validated the taxonomic classification and genomic abundance of various pathogens, reinforcing the reliability of the metagenomic approach for pathogen detection in wastewater and offering potential for pathogen-specific diagnostic applications.

Principal coordinate analysis (PCoA) based on Aitchison distance revealed marked dissimilarities in bacterial community structure across wastewater catchments serving low-, middle-, and high-income areas. At the genus level (n = 1126), variability in diversity and relative abundance among sites ranged from 10.7% to 45.7%, while at the species level (n = 3867), variability ranged from 13.7% to 32.7% (Figure 4), indicating substantial stratification of microbial communities by socioeconomic context. These findings are consistent with previous work showing that wastewater microbiomes and resistomes reflect local demographic, environmental, and infrastructural conditions [5,10]. Distinct taxonomic signatures further differentiated income strata. *Fibrobacter* spp. was most abundant in low-income areas, suggesting a microbial signature linked to environmental exposures, diet, or sanitation conditions characteristic of these communities [18]. Middle-income areas were characterized by higher relative abundances of *Candidafus*, *Tolumonas*, *Gynuella*, and *Hydrocarboniclastica*, whereas *Pseudomonas* spp. dominated the bacterial communities in high-income catchments (Figure 4). Such patterns underscore the influence of socioeconomic conditions on wastewater microbiome composition and highlight the potential of wastewater-based epidemiology to capture community-level microbial and functional differences [1,5].

Our findings from AMR analysis demonstrate that socioeconomic context is significantly associated with variation in wastewater resistome composition at the AMR gene class level, with socioeconomic category explaining approximately 10–14% of the variance after adjusting for sampling week and sequencing batch. This effect was robust to the choice of distance metric, being consistently observed using both Bray–Curtis dissimilarity and Robust Aitchison distance, the latter of which models the compositional structure of microbiome and resistome data [9]. These results align with prior work showing that AMR profiles in wastewater reflect local demographic and environmental conditions, including patterns of antimicrobial use, infrastructure, and sanitation [1,10,19].

The inconsistent signal observed at the drug class level—significant with Bray–Curtis but not with Robust Aitchison distance—suggests that aggregation to broader drug class categories may mask more subtle, but systematic, differences at the AMR gene class level. The lack of concordance between metrics, and the weaker effect sizes for Robust Aitchison distance at the drug class level (R^2^ ≈ 0.03–0.04, *p* = 0.32–0.37), may reflect the disproportionate influence of highly abundant, ubiquitous drug classes that dominate the resistome across catchments and dilute socioeconomic contrasts. Similar challenges in interpreting higher-level functional aggregations of resistome data have been reported in other wastewater-based and environmental surveillance studies [20,21].

Post hoc pairwise comparisons further indicate that the middle-income catchments primarily drive the observed socioeconomic signal at the AMR gene class level, differing significantly from each of the other categories, while low-income, high-income, and mixed/composite sites were not distinguishable. This pattern may reflect an intersection of factors typical of transitioning urban environments, such as intermediate but heterogeneous access to healthcare, variable antimicrobial usage and stewardship practices, and partially developed sanitation and wastewater infrastructure [22,23]. In contrast, both higher-income areas with more regulated antimicrobial use and lower-income settings with potentially lower formal antibiotic consumption but differing exposure pathways may converge on more similar resistome profiles in wastewater, as has been suggested in comparative international surveys [5,10]. Notably, colistin resistance showed an ordination pattern distinct from all other antibiotic classes and appeared more characteristic of high-income catchments, warranting further investigation given colistin’s role as a last-resort antibiotic [23].

Collectively, these results highlight that socioeconomic context can shape resistome composition in urban wastewater, but that this influence is scale-dependent and most evident at finer AMR gene class resolution. They underscore the importance of using compositionally aware methods and multiple distance metrics when interpreting resistome data and suggest that middle-income settings may represent critical targets for AMR surveillance and intervention. Future work integrating antimicrobial usage data, clinical resistance patterns, and infrastructure indicators will be essential to disentangle the specific drivers underlying these socioeconomic gradients in wastewater resistomes [8,22].

The proximity of bacterial pathogens and AMR genes in wastewater creates opportunities for the transfer of ARGs between environmental and pathogenic bacteria [6]. Routine and sustained wastewater surveillance is crucial for monitoring AMR activity in environmental, wildlife, livestock, and human interfaces [24]. The *Enterobacter* genus, one of the most abundant in Nairobi’s wastewater system, is linked to several AMR gene families, including multidrug resistance (MDR). The detection of these ARGs in the sewerage system warrants public health attention, especially considering the potential transfer to pathogenic Gram-positive enterococci or opportunistic pathogens such as *Staphylococcus aureus*.

We acknowledge limitations in this study. Although clinically relevant pathogens and AMR genes were detected, corresponding clinical data were not available for direct comparison with wastewater findings. In addition, metagenomic sequencing provides relative rather than absolute abundance estimates, and socio-economic status was inferred at the site level. Nevertheless, these limitations do not detract from the study’s contribution, which demonstrates the value of wastewater surveillance for monitoring population-level public health risks and provides an important baseline for future integrated surveillance efforts.

In conclusion, this study demonstrates that wastewater metagenomics can effectively detect and quantify multidrug-resistant organisms, including ESKAPE pathogens and other clinically relevant bacteria, within urban sewerage systems. Beyond mere detection, our analysis reveals that resistance gene and pathogen diversity patterns can be extrapolated to map socioeconomic disparities, with low-income areas exhibiting significantly higher variability. These results establish wastewater-based surveillance as a powerful, scalable public health intelligence tool—capable not only of monitoring emerging threats but also of informing targeted, equity-driven policy interventions in resource-limited settings.

## 4. Materials and Methods

### 4.1. Study Design

Wastewater samples were longitudinally collected from ten closed sewerage network sites across Nairobi, Kenya (Figure 1). Grab sampling (1 litre) was performed three times per week (Tuesday, Wednesday, Thursday between 6.00 to 8 am) over a three-month period to capture temporal dynamics in microbial communities. All samples were transported under controlled conditions to the International Livestock Research Institute (ILRI) laboratories for nucleic acid extraction and subsequent shotgun metagenomic sequencing.

### 4.2. Mapping Nairobi Metropolitan Sewerage Network

The Nairobi metropolitan sewerage network was mapped to guide strategic selection of sampling sites representing diverse socio-economic and geographical catchments (Table 1). Sampling locations were categorised into three strata: high-income suburbs, middle-income residential areas, and low-income informal settlements. Dandora, the city’s primary wastewater treatment plant (WWTP), served as the central point where all the sewer networks covered in this study converge for treatment.

### 4.3. Total Nucleic Acid Extraction and Library Preparation

Total nucleic acids were extracted using the Wizard^®^ Enviro Total Nucleic Acid Kit (Promega, Madison, WI, USA). The extracted material was treated with RNase A (Qiagen, Hilden, Germany) to remove RNA, and DNA was used as input for Illumina library construction (Illumina, Inc., San Diego, CA, USA). Libraries were prepared and sequenced using the NextSeq platform at the ILRI Genomics Platform, Nairobi, Kenya (https://www.ilri.org/genomics-platform, accessed on 1 August 2026).

### 4.4. Metagenomic Sample Sequencing

A total of 156 wastewater samples were collected from 10 sewerage sites across Nairobi and analyzed using nucleic acid sequencing to capture the metagenomic content. Of these, 89 samples underwent shotgun metagenomic sequencing and analysis, yielding high-quality datasets with a mean of 20,444,063 reads (Q ≥ 20) per sample, and averaging 12,702,489 taxonomically classifiable bacterial reads. The sequence data is available in the NCBI Sequence Read Archive (SRA) under BioProject accession number PRJNA1048855.

### 4.5. Sequence Data Analysis Using Metagenomic Bioinformatic Workflow

We performed metagenomic analysis using the nf-core/mag pipeline (v2.2.1) implemented in Nextflow [25,26]. Raw reads were quality-filtered (Q ≥ 20) and adapter-trimmed using *fastp* (v0.23.2) [27]. Reads aligning to the human reference genome were removed using *Bowtie2* (v2.4.2) [28]. Taxonomic classification was done with *Centrifuge* (v1.0.4, default options) [29] against a custom database comprising non-redundant NCBI’s nt database (2024/02/22—human, bacteria, protist, virus, fungi and archaea). All downstream analyses were performed in Rstudio implemented within the statistical programming language R [30]. Taxonomic abundance tables were merged, filtered, and visualized using *Pavian* (v2.6.2) and tidyverse (v2.0.0) [31,32]. PCoA was conducted using the Aitchison distance following centered log-ratio (CLR) transformation. Alpha diversity metrics and rarefaction curves were calculated using the *vegan* package (v2.6) in R (v4.2.1) [33].

### 4.6. Resistome Prediction

Antibiotic resistance genes were identified by aligning quality-trimmed sequencing reads against the ResFinder database (v2.4.0) [34] using ResFinder software (v4.6.0) [35]. A minimum threshold of 90% sequence identity and coverage (90%) was applied to match assemblies to reference sequences. The relative abundance of ARGs was calculated by normalizing ARG counts to the number of quality-trimmed reads per sample. Identified ARGs were categorized according to antibiotic drug classes, and their distribution was analysed in relation to the socio-economic status of the sampling sites. ARGs’ abundance datasets were ordinated by Robust Aitchison distance using both PCoA and non-metric multidimensional scaling (NMDS), with 95% confidence ellipses drawn around socioeconomic categories using the *vegan* and *stats* packages in R (v4.2.1) [33]. To test whether socioeconomic category affected variance in overall AMR community composition across all 4 groups, we performed PERMANOVA analysis, and post hoc pairwise Adonis was conducted to determine which group drove the variance using the *pairwiseAdonis* package (v0.4.1).

## Figures and Tables

**Table 1:** Socio-Economic Classification of Sampling Sites in Nairobi, Kenya.

Sampling Site	Administrative Zone (Sub-County)	Socio-Economic Status (Income)	Samples Collected (Weekly Frequency)	Samples Sequenced/Analysed	Total Population *
Dandora	Njiru	Central-WWTP	41 (3)	22	623,471
Donholm	Embakasi	Middle	13 (1)	8	983,232
Fedha	Embakasi	Middle	13 (1)	8	983,232
Githurai	Kasarani	Low	13 (1)	6	772,586
Kibera	Kibra	Low	12 (1)	7	181,509
King’ara Road	Westlands	High	13 (1)	8	301,295
Majengo	Kamukunji	Low	12 (1)	9	263,462
Mathare	Mathare	Low	13 (1)	6	204,469
Muthaiga	Westlands	High	13 (1)	8	301,295
Utawala	Njiru	Middle	13 (1)	7	623,471

* Population estimates were obtained from the Kenya National Bureau of Statistics (KNBS) 2019 Kenya Population and Housing Census. The Dandora sampling site is a convergence point for multiple sewer lines; therefore, the reported population does not reflect the total population contributing to the sampled wastewater.

## Data Availability

Wastewater genomic sequence data generated have been submitted to the National Center for Biotechnology Information (NCBI, https://www.ncbi.nlm.nih.gov/) for free access under BioProject PRJNA1048855.

## Supplementary Materials

The following supporting information can be downloaded at: https://www.mdpi.com/article/10.3390/antibiotics15090845/s1, Figure S1: Correlation analysis between qPCR ct-values and genomic abundance of *Vibrio cholerae* and *Klebsiella pneumoniae*; Figure S2: Drug Classes NMDS Ordinations (Robust-Aitchison distance of Relative abundance) for all 11 drug classes detected; Figure S3: Spatiotemporal relative abundance of selected bacterial pathogens across sampling sites and time points in Nairobi, Kenya: The Dandora sampling site; Table S1: qPCR validation primers [36,37]; Table S2: Top 50 Relative abundance ARGs.
