# Supplementary material for: Wastewater Metagenomic Surveillance Reveals Socioeconomic Patterns of Pathogen Diversity and Antimicrobial Resistance in Nairobi, Kenya
Source: Antibiotics (Basel). 2026 Aug 31;15(9):845. doi: 10.3390/antibiotics15090845 (PMC13603338; doi:10.3390/antibiotics15090845)
Supplement: Supplementary file 1 [file antibiotics-15-00845-s001.zip › antibiotics-4483659-supplementary.pdf]

Wastewater Metagenomic Surveillance Reveals Socioeconomic Patterns of Pathogen Diversity and Antimicrobial Resistance in Nairobi, Kenya

Benard Mware [1], Gilbert Kibet [1], Kennedy Mwangi [1], Doreen Lugano [1], Shebbar Osiany [1], Edward Kiritu [1], Paul Dobi [1], Collins Muli [1], John Juma [1], Regina Njeru [1], Eunice M. Machuka [1], Bernard Bett [1], Ahmed Ogwell [2], Edward O. Abworo [1], Tulio de Oliveira [3], Dishon M Muloi [1] and Sofonías K. Tessema [2] & Samuel O. Oyola [1*]

a) Figure S1: Correlation analysis between qPCR ct-values and genomic abundance of *Vibrio cholerae* and *Klebsiella pneumoniae*

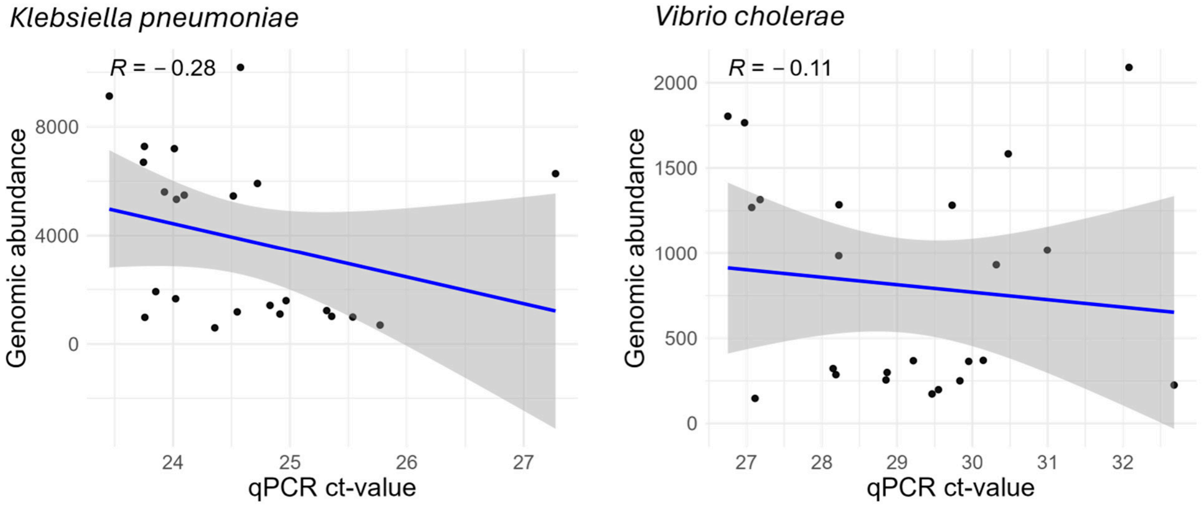

b) Figure S2: Drug Classes NMDS Ordinations (Robust-Aitchison distance of Relative abundance) for all 11 drug classes detected.

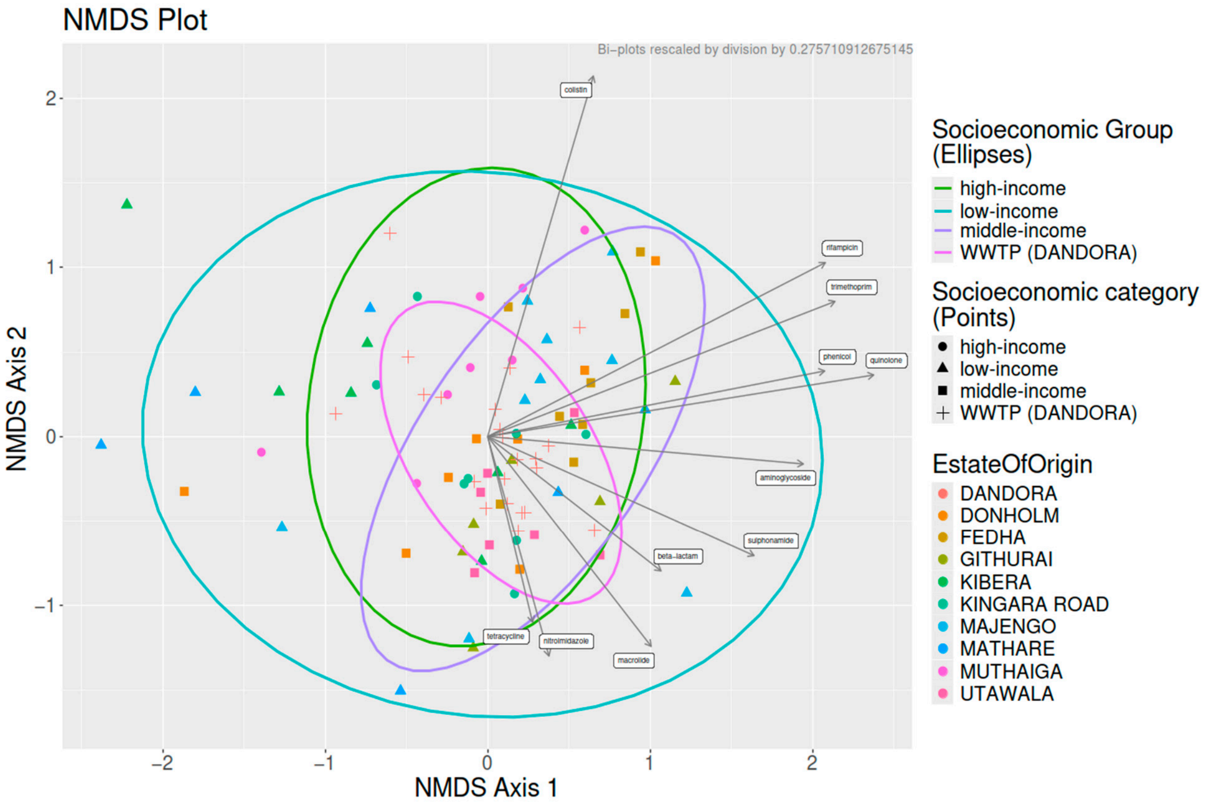

c) **Figure S3.** Spatiotemporal relative abundance of selected bacterial pathogens across sampling sites and time points in Nairobi, Kenya. The Dandora sampling site, located at the Nairobi City wastewater treatment plant (WWTP), receives wastewater from multiple sewer lines and therefore exhibits a composite pathogen profile representing the cumulative microbial signatures across the entire catchment area. Temporal fluctuations in pathogen abundance reflect changes in the aggregated bacterial community over the study period, hence less fluctuations pathogen profiles compare to the single sewer line sites shown in Figure 5.

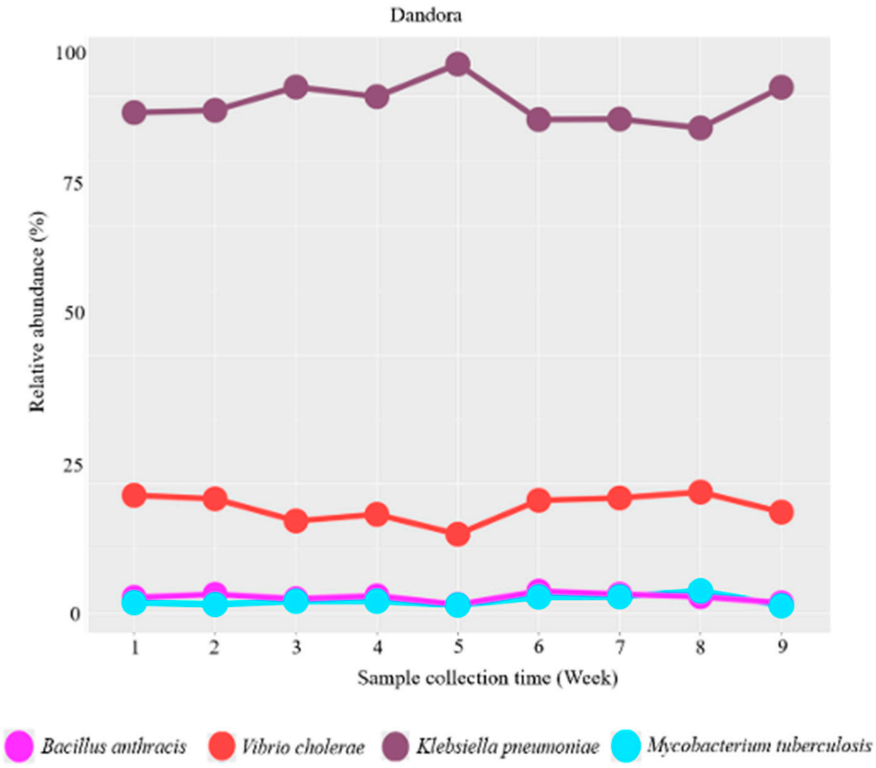

Table S1. qPCR validation primers

Pathogen	Gene	Primer Sequence	Reference
Vibrio cholerae	VCA0656	F: 5’ CAGTTTCTAACCGGGACTCAG 3’	https://doi.org/10.1093/femsle/fny156
		R: 5’ CCTTGCCCGACACAATTTG 3’	
		Probe: 5’ (6FAM) AGAGGGATCTGGAAGTCAGCTATCGC (BHQ1) 3’	
Klebsiella pneumoniae	KP-F	CGATGCTACTTATCCCGACA	https://doi.org/10.1128/spectrum.04249-22
	KP-R	AGCCGGTTGAGACGTAAAC	
	KP-probe	6FAM-CCGATTGAAAAACGCTCCGGGC-BHQ1	

Table S2: Top 50 Relative abundance ARGs

ARGs	DANDORA	DONHOLM	FEDHA	GITHURAI	KIBERA	KINGARA ROAD	MAJENGO	MATHARE	MUTHAIGA	UTAWALA
ARR-3	1.19E-06	2.53E-06	4.48E-06	2.19E-06	7.84E-07	1.20E-06	1.12E-06	1.67E-06	1.83E-06	1.81E-06
aac(6')-aph(2'')	2.39E-06	2.27E-06	2.66E-06	2.17E-06	1.36E-06	2.02E-06	1.86E-06	2.42E-06	2.04E-06	4.31E-06
aadA2	2.08E-06	2.56E-06	2.85E-06	2.24E-06	1.16E-06	1.80E-06	1.62E-06	1.40E-06	1.89E-06	2.88E-06
ant(2'')-Ia	1.39E-06	2.31E-06	3.74E-06	1.69E-06	6.34E-07	1.43E-06	1.22E-06	1.73E-06	1.27E-06	1.90E-06
ant(3'')-Ia	6.18E-06	1.06E-05	1.64E-05	9.10E-06	3.46E-06	5.05E-06	7.08E-06	6.16E-06	7.43E-06	8.46E-06
ant(6)-Ia	1.44E-06	2.60E-06	1.52E-06	1.91E-06	1.52E-06	1.55E-06	1.85E-06	5.06E-06	2.02E-06	2.14E-06
aph(3'')-Ib	3.97E-06	4.60E-06	6.70E-06	5.60E-06	2.82E-06	3.30E-06	4.19E-06	5.31E-06	3.96E-06	5.98E-06
aph(6)-Id	4.00E-06	5.09E-06	6.40E-06	5.31E-06	2.37E-06	3.34E-06	4.38E-06	4.45E-06	3.59E-06	6.21E-06
blaCMY-4	1.53E-06	1.91E-06	2.03E-06	2.67E-06	6.56E-07	1.56E-06	1.27E-06	2.58E-06	9.67E-07	1.87E-06
blaGES-1	2.43E-06	3.79E-06	7.53E-06	3.80E-06	8.99E-07	2.41E-06	1.75E-06	2.41E-06	6.03E-06	1.09E-06
blaOXA-10	4.20E-06	7.33E-06	1.08E-05	5.76E-06	2.19E-06	4.13E-06	5.06E-06	3.34E-06	5.80E-06	6.08E-06
blaOXA-347	5.24E-06	4.45E-06	4.36E-06	5.10E-06	2.09E-06	3.31E-06	2.32E-06	3.39E-06	2.85E-06	5.08E-06
blaOXA-490	2.37E-06	2.80E-06	2.68E-06	2.05E-06	2.35E-06	1.89E-06	3.05E-06	1.29E-06	2.94E-06	4.02E-06
blaVEB-1	1.47E-05	1.89E-05	2.13E-05	1.44E-05	7.22E-06	9.16E-06	1.28E-05	7.53E-06	1.19E-05	1.32E-05
catQ	1.89E-06	2.11E-06	1.72E-06	1.68E-06	1.99E-06	1.67E-06	1.44E-06	3.86E-06	9.09E-07	2.15E-06
cfxA	9.35E-07	2.81E-06	2.74E-06	2.19E-06	9.57E-07	1.35E-06	1.43E-06	2.44E-06	1.52E-06	1.67E-06
cfxA3	1.68E-05	5.22E-05	4.70E-05	4.14E-05	1.31E-05	2.71E-05	2.81E-05	4.14E-05	3.32E-05	3.33E-05
cfxA5	9.99E-07	3.04E-06	2.59E-06	2.42E-06	1.66E-06	4.42E-06	1.08E-06	2.00E-06	1.59E-06	1.70E-06

cfxA6	1.50E-05	2.65E-05	2.35E-05	3.39E-05	1.23E-05	1.67E-05	3.41E-05	4.36E-05	3.79E-05	2.11E-05
cmlA1	3.74E-06	8.28E-06	1.15E-05	6.55E-06	1.54E-06	3.93E-06	4.82E-06	3.91E-06	4.48E-06	6.46E-06
dfrA1	1.75E-06	3.69E-06	5.41E-06	3.04E-06	1.25E-06	2.33E-06	2.46E-06	2.54E-06	1.72E-06	2.49E-06
ere(A)	2.85E-06	1.97E-06	1.62E-06	3.44E-06	3.05E-06	2.33E-06	2.20E-06	2.12E-06	1.33E-06	4.85E-06
ere(B)	2.59E-06	3.23E-06	1.78E-06	1.42E-06	1.68E-06	1.56E-06	1.54E-06	1.50E-06	9.49E-07	2.56E-06
ere(D)	5.32E-06	4.75E-06	5.06E-06	5.94E-06	3.05E-06	3.95E-06	3.24E-06	3.59E-06	3.99E-06	7.10E-06
erm(F)	3.77E-05	7.94E-05	5.92E-05	5.75E-05	2.05E-05	4.71E-05	4.03E-05	3.82E-05	4.98E-05	6.17E-05
mef(A)	1.52E-05	2.85E-05	1.73E-05	1.33E-05	8.69E-06	1.13E-05	1.12E-05	1.26E-05	1.52E-05	1.82E-05
mef(B)	3.17E-06	2.26E-06	1.55E-06	2.45E-06	3.13E-06	2.13E-06	2.67E-06	4.68E-06	7.49E-07	2.33E-06
mef(C)	2.44E-06	1.48E-06	1.28E-06	1.95E-06	1.39E-06	9.83E-07	1.30E-06	1.19E-06	2.50E-06	2.67E-06
mph(A)	3.07E-06	7.00E-06	1.03E-05	5.46E-06	1.15E-06	3.69E-06	2.89E-06	3.85E-06	3.33E-06	6.79E-06
mph(E)	6.96E-06	1.13E-05	1.57E-05	8.86E-06	3.54E-06	5.87E-06	5.07E-06	8.02E-06	8.69E-06	1.40E-05
msr(D)	2.57E-05	3.68E-05	2.74E-05	1.90E-05	1.59E-05	1.94E-05	2.00E-05	1.84E-05	1.74E-05	3.44E-05
msr(E)	1.01E-05	1.60E-05	2.29E-05	1.44E-05	4.28E-06	8.61E-06	7.40E-06	1.09E-05	1.30E-05	2.09E-05
nimE	1.50E-05	4.64E-05	3.21E-05	3.12E-05	8.52E-06	2.07E-05	1.21E-05	1.68E-05	1.44E-05	3.35E-05
qnrS2	2.62E-06	4.10E-06	6.55E-06	5.39E-06	8.92E-07	2.75E-06	2.35E-06	4.18E-06	3.39E-06	4.17E-06
qnrVC1	1.30E-06	2.76E-06	5.18E-06	3.20E-06	4.47E-07	1.13E-06	1.72E-06	1.13E-06	2.94E-06	1.87E-06
qnrVC4	1.90E-06	3.62E-06	6.58E-06	2.90E-06	6.22E-07	2.09E-06	1.64E-06	1.81E-06	2.23E-06	3.32E-06
sul1	1.03E-05	1.64E-05	2.34E-05	1.57E-05	5.73E-06	9.33E-06	1.12E-05	1.14E-05	1.02E-05	1.58E-05
sul2	1.29E-05	3.25E-05	1.99E-05	2.05E-05	9.19E-06	1.55E-05	1.29E-05	1.85E-05	1.10E-05	2.29E-05
tet(32)	2.13E-06	2.26E-06	1.71E-06	2.22E-06	2.68E-06	1.61E-06	2.48E-06	6.30E-06	2.43E-06	2.91E-06
tet(36)	7.70E-06	1.13E-05	1.82E-05	1.47E-05	2.43E-06	6.87E-06	2.81E-06	4.36E-06	7.34E-06	1.38E-05
tet(39)	1.76E-06	1.57E-06	3.00E-06	2.10E-06	1.26E-06	1.20E-06	1.64E-06	1.85E-06	1.69E-06	3.99E-06
tet(40)	3.11E-06	3.14E-06	2.06E-06	2.87E-06	2.60E-06	1.73E-06	4.87E-06	6.24E-06	3.87E-06	2.71E-06
tet(A)	5.33E-06	9.46E-06	1.23E-05	7.39E-06	4.41E-06	4.79E-06	4.99E-06	6.00E-06	4.04E-06	7.82E-06
tet(C)	7.98E-06	7.76E-06	8.49E-06	8.19E-06	3.63E-06	4.77E-06	4.83E-06	2.94E-06	4.27E-06	1.20E-05
tet(M)	7.19E-06	5.72E-06	4.92E-06	6.30E-06	4.74E-06	5.28E-06	4.45E-06	1.22E-05	2.84E-06	5.83E-06
tet(O)	6.39E-06	6.48E-06	3.77E-06	5.74E-06	5.35E-06	3.40E-06	8.38E-06	1.19E-05	6.26E-06	4.22E-06
tet(O/32/O)	4.61E-06	3.47E-06	3.68E-06	3.31E-06	4.64E-06	3.36E-06	6.29E-06	1.27E-05	4.79E-06	4.04E-06
tet(Q)	6.19E-05	0.000149419	0.000123602	0.000120645	4.36E-05	8.62E-05	0.000106451	0.000127772	0.000127381	0.00010544
tet(W)	9.66E-06	1.32E-05	1.14E-05	1.35E-05	1.07E-05	1.14E-05	1.49E-05	3.38E-05	1.59E-05	1.25E-05
tet(X)	8.57E-06	2.88E-05	2.25E-05	1.25E-05	3.83E-06	1.26E-05	8.45E-06	6.27E-06	1.40E-05	2.06E-05
